# Supplementary material for: Occupational Branch and Labor Market Marginalization among Young Employees with Adult Onset of Attention Deficit Hyperactivity Disorder—A Population-Based Matched Cohort Study
Source: Int J Environ Res Public Health. 2022 Jun 14;19(12):7254. doi: 10.3390/ijerph19127254 (PMC9223828; doi:10.3390/ijerph19127254)
Supplement: Supplementary file 1 [file ijerph-19-07254-s001.zip › ijerph-1744603-supplementary.pdf]

Supplement material

Supplement table S1: STROBE Statement—Checklist of items that should be included in reports of ***cohort studies***

	Item No	Recommendation	Page No
**Title and abstract**	1	(*a*) Indicate the study's design with a commonly used term in the title or the abstract	1
		(*b*) Provide in the abstract an informative and balanced summary of what was done and what was found	1
**Introduction**			
Background/rationale	2	Explain the scientific background and rationale for the investigation being reported	2
Objectives	3	State specific objectives, including any prespecified hypotheses	2
**Methods**			
Study design	4	Present key elements of study design early in the paper	2
Setting	5	Describe the setting, locations, and relevant dates, including periods of recruitment, exposure, follow-up, and data collection	3
Participants	6	(*a*) Give the eligibility criteria, and the sources and methods of selection of participants. Describe methods of follow-up	3
		(*b*) For matched studies, give matching criteria and number of exposed and unexposed	2
Variables	7	Clearly define all outcomes, exposures, predictors, potential confounders, and effect modifiers. Give diagnostic criteria, if applicable	3
Data sources/ measurement	8*	For each variable of interest, give sources of data and details of methods of assessment (measurement). Describe comparability of assessment methods if there is more than one group	2
Bias	9	Describe any efforts to address potential sources of bias	2
Study size	10	Explain how the study size was arrived at	2
Quantitative variables	11	Explain how quantitative variables were handled in the analyses. If applicable, describe which groupings were chosen and why	3
Statistical methods	12	(*a*) Describe all statistical methods, including those used to control for confounding	3
		(*b*) Describe any methods used to examine subgroups and interactions	3

		(*c*) Explain how missing data were addressed	3
		(*d*) If applicable, explain how loss to follow-up was addressed	2
		(*e*) Describe any sensitivity analyses	3
**Results**			
Participants	13*	(a) Report numbers of individuals at each stage of study—eg numbers potentially eligible, examined for eligibility, confirmed eligible, included in the study, completing follow-up, and analysed	2,4
		(b) Give reasons for non-participation at each stage	NA
		(c) Consider use of a flow diagram	
Descriptive data	14*	(a) Give characteristics of study participants (eg demographic, clinical, social) and information on exposures and potential confounders	4
		(b) Indicate number of participants with missing data for each variable of interest	NA
		(c) Summarise follow-up time (eg, average and total amount)	NA
Outcome data	15*	Report numbers of outcome events or summary measures over time	4

Main results	16	(*a*) Give unadjusted estimates and, if applicable, confounder-adjusted estimates and their precision (eg, 95% confidence interval). Make clear which confounders were adjusted for and why they were included	4, table 2
		(*b*) Report category boundaries when continuous variables were categorized	4
		(*c*) If relevant, consider translating estimates of relative risk into absolute risk for a meaningful time period	NA
Other analyses	17	Report other analyses done—eg analyses of subgroups and interactions, and sensitivity analyses	Page 6; Table 3,4
**Discussion**			
Key results	18	Summarise key results with reference to study objectives	8

Limitations	19	Discuss limitations of the study, taking into account sources of potential bias or imprecision. Discuss both direction and magnitude of any potential bias	9
Interpretation	20	Give a cautious overall interpretation of results considering objectives, limitations, multiplicity of analyses, results from similar studies, and other relevant evidence	8,9
Generalisability	21	Discuss the generalisability (external validity) of the study results	9
**Other information**			
Funding	22	Give the source of funding and the role of the funders for the present study and, if applicable, for the original study on which the present article is based	9

*Give information separately for exposed and unexposed groups.

Note: An Explanation and Elaboration article discusses each checklist item and gives methodological background and published examples of transparent reporting. The STROBE checklist is best used in conjunction with this article (freely available on the Web sites of PLoS Medicine at http://www.plosmedicine.org/, Annals of Internal Medicine at http://www.annals.org/, and Epidemiology at http://www.epidem.com/). Information on the STROBE Initiative is available at http://www.strobe-statement.org.

Supplement Table S2. Distribution of study characteristics by occupational branches

	Manufacturing (n=7755)		Construction (n=7348)		Trade and communication (n=15,389)		Financial and business service (n=6457)		Education and research (n=3641)		Health and social care (n=12,628)		Other (n=13,112)	
	N	%	N	%	N	%	N	%	N	%	N	%	N	%
Sex														
Female	1276	16.5	506	6.9	6798	44.2	2475	38.3	2662	73.1	9713	76.9	6677	50.9
Male	6479	83.5	6842	93.1	8591	55.8	3982	61.7	979	26.9	2915	23.1	6435	49.1
Years of education														
<10	991	12.8	967	13.2	1836	11.9	660	10.2	206	5.7	1131	9.0	1438	11.0
10-12	5371	69.3	5821	79.2	10,571	68.7	3285	50.9	1704	46.8	7088	56.1	7356	56.1
>12	1393	18.0	560	7.6	2982	19.4	2512	38.9	1731	47.5	4409	34.9	4318	32.9
Country of origin														
Sweden	7011	90.4	6864	93.4	13,700	89.0	5663	87.7	3222	88.5	10,934	86.6	11,463	87.4
Nordic countries	34	0.4	37	0.5	91	0.6	49	0.8	23	0.6	76	0.6	87	0.7
Other European country	128	1.7	185	2.5	225	1.5	131	2.0	80	2.2	201	1.6	287	2.2
Other	582	7.5	262	3.6	1373	8.9	614	9.5	316	8.7	1417	11.2	1275	9.7
Family status														
Married/cohabiting without children	184	2.4	117	1.6	403	2.6	204	3.2	148	4.1	493	3.9	421	3.2
Married/cohabiting with children	1012	13.0	1055	14.4	1809	11.8	619	9.6	553	15.2	2286	18.1	1323	10.1
Single without children	6491	83.7	6134	83.5	12,975	84.3	5546	85.9	2842	78.1	9326	73.9	11,142	85.0
Single with children	68	0.9	42	0.6	202	1.3	88	1.4	98	2.7	523	4.1	226	1.7
Living region														
Large cities	1771	22.8	2481	33.8	6688	43.5	3362	52.1	1707	46.9	4348	34.4	5984	45.6
Medium-sized cities	2915	37.6	2635	35.9	5414	35.2	2029	31.4	1220	33.5	4940	39.1	4400	33.6
Rural areas	3069	39.6	2232	30.4	3287	21.4	1066	16.5	714	19.6	3340	26.4	2728	20.8
Income quantile														

First	1103	14.2	1128	15.4	3614	23.5	1828	28.3	1011	27.8	3837	30.4	4080	31.1
Second	1310	16.9	1239	16.9	4035	26.2	1608	24.9	1144	31.4	3741	29.6	3496	26.7
Third	2074	26.7	1799	24.5	4221	27.4	1341	20.8	1026	28.2	3341	26.5	2787	21.3
Fourth	3268	42.1	3182	43.3	3519	22.9	1680	26.0	460	12.6	1709	13.5	2749	21.0
Blue/White collar worker														
Blue collar	5526	83.7	5676	92.6	11,475	87.0	2896	54.0	1885	55.1	9215	77.5	7213	66.7
White collar	1074	16.3	454	7.4	1715	13.0	2465	46.0	1536	44.9	2670	22.5	3593	33.3
Type of work														
Employed	7671	98.9	6944	94.5	15,079	98.0	6210	96.2	3621	99.5	12,594	99.7	12,193	93.0
Self-employed	84	1.1	404	5.5	310	2.0	247	3.8	20	0.5	34	0.3	919	7.0
Sickness absence one year prior to baseline														
No	7067	91.1	6698	91.2	14,087	91.5	5961	92.3	3312	91.0	11,268	89.2	12,124	92.5
Yes	688	8.9	650	8.8	1302	8.5	496	7.7	329	9.0	1360	10.8	988	7.5
Depression/bipolar disorders														
No	7481	96.5	7098	96.6	14,729	95.7	6200	96.0	3473	95.4	11,957	94.7	12,555	95.8
Yes	274	3.5	250	3.4	660	4.3	257	4.0	168	4.6	671	5.3	557	4.2
Anxiety/stress-related disorders														
No	7399	95.4	7061	96.1	14,621	95.0	6179	95.7	3439	94.5	11,856	93.9	12,445	94.9
Yes	356	4.6	287	3.9	768	5.0	278	4.3	202	5.5	772	6.1	667	5.1
Autism-spectrum disorders														
No	7700	99.3	7323	99.7	15,290	99.4	6411	99.3	3614	99.3	12,563	99.5	12,993	99.1
Yes	55	0.7	25	0.3	99	0.6	46	0.7	27	0.7	65	0.5	119	0.9
Substance abuse														
No	7529	97.1	7086	96.4	15,006	97.5	6292	97.4	3592	98.7	12,357	97.9	12,818	97.8
Yes	226	2.9	262	3.6	383	2.5	165	2.6	49	1.3	271	2.1	294	2.2
Behavioral/emotional disorders														
No	7727	99.6	7323	99.7	15,332	99.6	6427	99.5	3629	99.7	12,583	99.6	13,056	99.6
Yes	28	0.4	25	0.3	57	0.4	30	0.5	12	0.3	45	0.4	56	0.4
Schizophrenia/psychoses														

No	7736	99.8	7327	99.7	15,359	99.8	6440	99.7	3632	99.8	12,593	99.7	13,083	99.8
Yes	19	0.2	21	0.3	30	0.2	17	0.3	9	0.2	35	0.3	29	0.2
Other mental disorders														
No	7635	98.5	7237	98.5	15,065	97.9	6334	98.1	3554	97.6	12,259	97.1	12,807	97.7
Yes	120	1.5	111	1.5	324	2.1	123	1.9	87	2.4	369	2.9	305	2.3
Musculoskeletal disorders														
No	7263	93.7	6810	92.7	14,444	93.9	6093	94.4	3414	93.8	11,762	93.1	12,286	93.7
Yes	492	6.3	538	7.3	945	6.1	364	5.6	227	6.2	866	6.9	826	6.3
Other somatic disorders														
No	4774	61.6	4239	57.7	8785	57.1	3791	58.7	1824	50.1	6124	48.5	7378	56.3
Yes	2981	38.4	3109	42.3	6604	42.9	2666	41.3	1817	49.9	6504	51.5	5734	43.7

Supplement table S3. Labor market marginalization, sickness absence / disability pension and unemployment days by occupation branches

		**Manufacturing**	**Construction**	**Trade and communication**	**Financial and business service**	**Education and research**	**Health and social care**	**Other**
Mean days (standard deviation)	Labor market marginalization days	133 (286)	122 (267)	117 (270)	127 (280)	117 (262)	138 (296)	126 (278)
	SA/DP* days	59 (222)	59 (206)	63 (222)	59 (221)	76 (226)	94 (262)	65 (225)
	Unemployment days	74 (160)	63 (146)	54 (138)	68 (158)	41 (115)	44 (123)	60 (148)
Percentage of experiencing the outcome	Any labor market marginalization	50	50	46	45	50	54	48
	Any SA/DP* days	27	30	29	25	36	41	29
	Any unemployment days	35	32	28	31	25	25	31
Median days (interquartile range) among those who experiencing the outcome	Labor market marginalization days	114 (48, 269)	127 (55, 286)	110 (47, 271)	138 (55, 309)	102 (43, 215)	105 (43, 250)	116 (48, 287)
	SA/DP* days	70 (33, 177)	69 (31, 168)	73 (34, 179)	74 (31, 206)	82 (32, 178)	79 (34, 196)	74 (33, 194)
	Unemployment days	117 (55, 244)	135 (63, 264)	115 (53, 243)	132 (62, 267)	94 (46, 188)	100 (43, 213)	113 (53, 243)

*SA/DP sickness absence and disability pension

Supplement table S4. Labor market marginalization (LMM), sickness absence/ disability pension (SA/DP) and unemployment (UE) days in individuals with and without attention deficit hyperactivity disorders (ADHD) by occupational branches

	**Manufacturing**		**Construction**		**Trade and communication**		**Financial and business service**		**Education and research**		**Health and social care**		**Other**	
	Diagnosed by ADHD at baseline		Diagnosed by ADHD at baseline		Diagnosed by ADHD at baseline		Diagnosed by ADHD at baseline		Diagnosed by ADHD at baseline		Diagnosed by ADHD at baseline		Diagnosed by ADHD at baseline	
	**No**	**Yes**	**No**	**Yes**	**No**	**Yes**	**No**	**Yes**	**No**	**Yes**	**No**	**Yes**	**No**	**Yes**
Mean (SD days)														
LMM	94 (194)	516 (601)	88 (193)	460 (532)	84 (192)	452 (557)	89 (197)	505 (563)	87 (195)	416 (528)	100 (215)	514 (590)	93 (207)	453 (554)
SA/DP	30 (117)	350 (562)	35 (131)	302 (482)	37 (135)	324 (535)	29 (123)	354 (541)	52 (159)	314 (494)	61 (175)	423 (575)	40 (145)	316 (529)
UE	65 (148)	167 (229)	53 (132)	158 (226)	47 (128)	128 (196)	60 (147)	151 (225)	35 (103)	102 (190)	39 (116)	91 (172)	53 (138)	137 (212)
Percentage of individuals experiencing the outcome														
LMM	46	84	46	84	43	82	41	82	46	82	51	85	45	82
SA/DP	23	63	27	64	26	62	21	61	33	66	38	72	26	61
UE	32	63	29	64	25	58	28	61	23	53	23	48	28	59
**Mean days (standard deviation) among those who experiencing the outcome**														
LMM	203 (243)	617 (608)	190 (247)	549 (538)	196 (253)	551 (569)	214 (259)	618 (564)	188 (251)	508 (542)	195 (268)	608 (595)	207 (268)	555 (566)
SA/DP	127 (214)	553 (620)	130 (228)	469 (532)	142 (235)	519 (597)	139 (238)	582 (590)	157 (246)	478 (542)	159 (253)	586 (603)	153 (250)	515 (595)
UE	201 (201)	265 (240)	184 (189)	247 (241)	187 (198)	222 (214)	209 (212)	249 (243)	157 (166)	191 (225)	171 (191)	189 (207)	188 (205)	234 (232)

SD: standard deviation.

Supplement table S5. Associations between Attention Deficit Hyperactivity Disorder (ADHD) diagnosis and labour market marginalization (LMM). Incidence rate ratios (IRR) of LMM days and Odds Ratios (OR) of probability of >0 LMM days

Variables	**Labour market marginalization days**		**Sickness absence/ disability pension days**		**Unemployment days**	
	IRR (95% CI)	***IRR (95% CI)***	***IRR (95% CI)***	***IRR (95% CI)***	***IRR (95% CI)***	***IRR (95% CI)***
**ADHD diagnosis (ref: no)**						
Yes	1.48 (1.43–1.54)	1.48 (1.43–1.54)	1.70 (1.62 – 1.79)	1.71 (1.62 – 1.79)	1.13 (1.08 – 1.19)	1.13 (1.08 – 1.19)
Age (ref: 19-24)						
25-29	1.33 (1.30 – 1.36)	1.33 (1.30 – 1.37)	1.23 (1.19 – 1.27)	1.23 (1.19 – 1.27)	1.40 (1.36 – 1.45)	1.41 (1.36 – 1.45)
Gender (ref: female)						
Male	1.16 (1.13 – 1.19)	1.13 (1.10 – 1.16)	0.95 (0.92 – 0.99)	0.98 (0.94 – 1.01)	1.23 (1.19 – 1.27)	1.20 (1.16 – 1.24)
Years of education (ref: 0-9 years)						
10-12 years	0.78 (0.75 – 0.81)	0.78 (0.76 – 0.81)	0.84 (0.81 – 0.88)	0.84 (0.80 – 0.88)	0.81 (0.77 – 0.84)	0.81 (0.78 – 0.84)
>12 years	0.65 (0.62 – 0.68)	0.65 (0.63 – 0.68)	0.71 (0.67 – 0.75)	0.70 (0.66 – 0.74)	0.72 (0.69 – 0.76)	0.73 (0.69 – 0.77)
Country of origin (ref: Sweden)						
Nordic countries	0.98 (0.85 – 1.14)	0.98 (0.84 – 1.14)	1.04 (0.86 – 1.26)	1.04 (0.86 – 1.26)	1.08 (0.89 – 1.33)	1.08 (0.88 – 1.32)
European Union	1.34 (1.24 – 1.46)	1.34 (1.23 – 1.45)	1.15 (1.03 – 1.29)	1.15 (1.03 – 1.29)	1.37 (1.23 – 1.52)	1.36 (1.23 – 1.51)
Outside EU	1.33 (1.28 – 1.38)	1.33 (1.28 – 1.38)	1.04 (0.98 – 1.10)	1.04 (0.98 – 1.09)	1.38 (1.31 – 1.44)	1.38 (1.31 – 1.44)
Family status (ref: single without children)						
Single with children	1.13 (1.06 – 1.21)	1.14 (1.06 – 1.22)	1.12 (1.03 – 1.22)	1.12 (1.03 – 1.21)	1.07 (0.97 – 1.17)	1.08 (0.98 – 1.18)
Married or cohabitant without children	1.09 (1.02 – 1.16)	1.09 (1.02 – 1.16)	1.08 (0.99 – 1.17)	1.08 (0.99 – 1.17)	1.06 (0.97 – 1.16)	1.07 (0.97 – 1.16)
Married or cohabitant with children	0.94 (0.91 – 0.97)	0.94 (0.91 – 0.98)	0.93 (0.89 – 0.97)	0.93 (0.89 – 0.97)	1.00 (0.95 – 1.05)	1.00 (0.96 – 1.05)
Region (ref: large city)						
Medium-sized cities	1.04 (1.01 – 1.07)	1.04 (1.01 – 1.07)	1.04 (1.00 – 1.07)	1.04 (1.00 – 1.07)	1.01 (0.97 – 1.04)	1.00 (0.97 – 1.04)
Rural areas	1.04 (1.01 – 1.07)	1.04 (1.01 – 1.07)	1.04 (1.00 – 1.08)	1.04 (1.00 – 1.08)	0.98 (0.95 – 1.02)	0.98 (0.94 – 1.02)
Income quantile (ref: first)						
Second quantile	1.00 (0.97 – 1.03)	1.00 (0.97 – 1.03)	0.96 (0.92 – 1.00)	0.96 (0.92 – 1.00)	1.04 (1.00 – 1.08)	1.04 (1.00 – 1.08)
Third quantile	0.86 (0.83 – 0.89)	0.86 (0.83 – 0.89)	0.84 (0.81 – 0.88)	0.85 (0.81 – 0.88)	0.99 (0.95 – 1.03)	0.98 (0.94 – 1.02)
Fourth quantile	0.70 (0.67 – 0.72)	0.69 (0.67 – 0.72)	0.71 (0.68 – 0.75)	0.72 (0.68 – 0.76)	0.90 (0.85 – 0.94)	0.88 (0.84 – 0.93)

Blue/White collar worker (ref: blue)						
White collar	0.96 (0.93 – 0.99)	0.95 (0.91 – 0.98)	1.02 (0.97 – 1.07)	1.01 (0.97 – 1.06)	0.96 (0.91 – 1.01)	0.95 (0.90 – 1.00)
Type of work (ref: employee)						
Self-employed	0.97 (0.89 – 1.04)	0.95 (0.88 – 1.03)	1.05 (0.95 – 1.16)	1.05 (0.95 – 1.16)	1.02 (0.92 – 1.13)	1.00 (0.90 – 1.11)
SA before baseline (ref: no)						
Yes	1.44 (1.40 – 1.49)	1.44 (1.40 – 1.49)	1.54 (1.48 – 1.60)	1.55 (1.49 – 1.61)	1.01 (0.96 – 1.06)	1.01 (0.96 – 1.06)
Common mental disorders (ref no)						
Depression/bipolar disorders	1.50 (1.43 – 1.57)	1.49 (1.42 – 1.57)	1.57 (1.48 – 1.66)	1.57 (1.48 – 1.66)	1.00 (0.94 – 1.07)	1.00 (0.94 – 1.07)
Anxiety/stress-related disorders	1.39 (1.33 – 1.45)	1.39 (1.33 – 1.46)	1.47 (1.39 – 1.54)	1.47 (1.39 – 1.55)	0.99 (0.94 – 1.05)	0.99 (0.94 – 1.05)
Autism-spectrum disorders	1.46 (1.32 – 1.62)	1.45 (1.31 – 1.61)	1.66 (1.47 – 1.88)	1.67 (1.48 – 1.88)	1.05 (0.92 – 1.19)	1.05 (0.92 – 1.19)
Substance abuse	1.19 (1.12 – 1.26)	1.19 (1.12 – 1.26)	1.22 (1.14 – 1.31)	1.22 (1.14 – 1.31)	1.10 (1.03 – 1.18)	1.10 (1.03 – 1.18)
Behavioral/emotional disorders	1.06 (0.93 – 1.22)	1.06 (0.93 – 1.22)	1.08 (0.92 – 1.27)	1.07 (0.91 – 1.26)	1.02 (0.87 – 1.21)	1.02 (0.86 – 1.21)
Schizophrenia/psychoses	1.79 (1.52 – 2.10)	1.80 (1.53 – 2.11)	2.16 (1.81 – 2.59)	2.17 (1.81 – 2.60)	1.03 (0.84 – 1.26)	1.03 (0.84 – 1.26)
Other mental disorders	1.37 (1.29 – 1.45)	1.37 (1.29 – 1.45)	1.41 (1.32 – 1.52)	1.41 (1.32 – 1.52)	0.95 (0.88 – 1.03)	0.95 (0.87 – 1.02)
Somatic disorders (ref: no)						
Musculoskeletal disorders	1.13 (1.09 – 1.17)	1.13 (1.09 – 1.18)	1.21 (1.16 – 1.27)	1.21 (1.16 – 1.27)	0.98 (0.92 – 1.03)	0.98 (0.93 – 1.03)
Other somatic disorders	1.02 (1.00 – 1.05)	1.03 (1.00 – 1.05)	1.09 (1.06 – 1.13)	1.09 (1.06 – 1.13)	1.01 (0.98 – 1.04)	1.01 (0.98 – 1.04)
Occupational branch						
Construction	*Not included*	*Reference*	*Not included*	*Reference*	*Not included*	*Reference*
Manufacturing	*Not included*	1.05 (1.00 – 1.10)	*Not included*	0.92 (0.86 – 0.98)	*Not included*	1.10 (1.04 – 1.17)
Trade and communication	*Not included*	0.96 (0.92 – 1.00)	*Not included*	0.98 (0.92 – 1.04)	*Not included*	1.00 (0.95 – 1.05)
Financial and business services	*Not included*	1.08 (1.03 – 1.14)	*Not included*	0.97 (0.90 – 1.04)	*Not included*	1.13 (1.06 – 1.20)
Education and research	*Not included*	0.93 (0.87 – 0.99)	*Not included*	1.02 (0.94 – 1.10)	*Not included*	0.91 (0.84 – 0.99)
Health and social care	*Not included*	0.94 (0.90 – 0.99)	*Not included*	1.03 (0.97 – 1.10)	*Not included*	0.94 (0.88 – 0.99)
Other	*Not included*	1.03 (0.98 – 1.07)	*Not included*	1.01 (0.95 – 1.08)	*Not included*	1.06 (1.00 – 1.12)
	OR (95% CI)	***OR (95% CI)***	***OR (95% CI)***	***OR (95% CI)***	***OR (95% CI)***	***OR (95% CI)***
**ADHD diagnosis (ref:no)**						
Yes	2.36 (2.17-2.56)	2.36 (2.17-2.57)	1.94 (1.80-2.10)	1.95 (1.81-2.11)	2.21 (2.06-2.38)	2.21 (2.06-2.38)

Age (ref: 19-24)						
25-29	1.10 (1.05-1.14)	1.10 (1.06-1.15)	1.15 (1.10-1.20)	1.15 (1.09-1.20)	1.03 (0.98-1.07)	1.05 (1.00-1.09)
Gender (ref: female)						
Male	0.77 (0.74-0.80)	0.71 (0.68-0.74)	0.46 (0.44-0.48)	0.47 (0.45-0.49)	1.36 (1.31-1.42)	1.16 (1.11-1.21)
Years of education (ref: 0-9 years)						
10-12 years	0.66 (0.62-0.7)	0.66 (0.62-0.7)	0.76 (0.72-0.81)	0.76 (0.71-0.8)	0.65 (0.62-0.69)	0.66 (0.62-0.69)
>12 years	0.46 (0.43-0.49)	0.47 (0.43-0.5)	0.62 (0.57-0.67)	0.61 (0.57-0.66)	0.42 (0.39-0.45)	0.43 (0.40-0.46)
Country of origin (ref: Sweden)						
Nordic countries	0.92 (0.73-1.15)	0.92 (0.74-1.16)	0.95 (0.74-1.21)	0.96 (0.75-1.22)	0.89 (0.69-1.15)	0.90 (0.70-1.15)
European Union	1.23 (1.09-1.39)	1.21 (1.07-1.36)	1.06 (0.92-1.22)	1.05 (0.92-1.21)	1.32 (1.16-1.51)	1.29 (1.13-1.47)
Outside EU	1.65 (1.55-1.75)	1.69 (1.59-1.79)	1.05 (0.98-1.12)	1.05 (0.98-1.12)	2.01 (1.89-2.14)	2.1 (1.97-2.23)
Family status (ref: single without children)						
Single with children	1.46 (1.27-1.68)	1.45 (1.26-1.67)	1.50 (1.32-1.71)	1.46 (1.28-1.66)	1.14 (1.01-1.3)	1.18 (1.04-1.34)
Married/cohabitant without children	1.25 (1.13-1.39)	1.26 (1.14-1.4)	1.28 (1.15-1.42)	1.27 (1.14-1.41)	1.10 (0.98-1.23)	1.12 (1-1.25)
Married /cohabitant with children	1.04 (0.98-1.09)	1.02 (0.97-1.08)	1.39 (1.31-1.47)	1.36 (1.28-1.44)	0.78 (0.73-0.82)	0.78 (0.74-0.83)
Region (ref large cities)						
Medium-sized cities	1.31 (1.26-1.36)	1.29 (1.24-1.35)	1.09 (1.04-1.14)	1.07 (1.02-1.12)	1.45 (1.39-1.51)	1.45 (1.39-1.52)
Rural areas	1.53 (1.47-1.6)	1.49 (1.43-1.56)	1.12 (1.07-1.18)	1.09 (1.04-1.15)	1.73 (1.65-1.82)	1.71 (1.63-1.79)
Income quantile (ref: first)						
Second quantile	0.96 (0.92-1.01)	0.96 (0.91-1.01)	1.11 (1.05-1.17)	1.11 (1.05-1.17)	0.88 (0.83-0.92)	0.87 (0.82-0.91)
Third quantile	0.8 (0.76-0.84)	0.79 (0.75-0.83)	1.29 (1.22-1.36)	1.29 (1.22-1.36)	0.56 (0.53-0.59)	0.54 (0.51-0.57)
Fourth quantile	0.55 (0.52-0.58)	0.51 (0.48-0.54)	1.16 (1.09-1.24)	1.16 (1.09-1.24)	0.3 (0.28-0.32)	0.27 (0.26-0.29)
Blue/White collar worker (ref: blue collar)						
White collar	0.80 (0.76-0.84)	0.8 (0.76-0.84)	0.83 (0.78-0.88)	0.85 (0.81-0.91)	0.77 (0.73-0.82)	0.74 (0.7-0.79)
Type of work (ref: employeed)						
Self-employed	0.46 (0.41-0.51)	0.44 (0.39-0.49)	0.83 (0.73-0.93)	0.84 (0.74-0.95)	0.35 (0.31-0.4)	0.32 (0.28-0.36)
SA before baseline (ref: no)						
Yes	8.67 (7.85-9.57)	8.67 (7.85-9.57)	3.74 (3.5-3.99)	3.74 (3.5-4)	1.37 (1.28-1.47)	1.36 (1.27-1.45)

Common mental disorders (ref: no)						
Depression/bipolar disorders	1.76 (1.55-2-00)	1.77 (1.56-2.02)	1.98 (1.78-2.21)	1.98 (1.78-2.21)	1.24 (1.12-1.37)	1.26 (1.14-1.39)
Anxiety/stress-related disorders	1.54 (1.38-1.73)	1.54 (1.38-1.73)	1.79 (1.63-1.98)	1.80 (1.63-1.98)	1.17 (1.06-1.28)	1.17 (1.07-1.29)
Autism-spectrum disorders	1.49 (1.11-2.02)	1.51 (1.12-2.04)	1.58 (1.24-2.02)	1.62 (1.27-2.07)	1.15 (0.93-1.41)	1.14 (0.92-1.4)
Substance abuse	1.63 (1.40-1.90)	1.62 (1.38-1.89)	1.31 (1.15-1.49)	1.30 (1.14-1.48)	1.35 (1.2-1.51)	1.34 (1.19-1.5)
Behavioral/emotional disorders	0.94 (0.66-1.34)	0.94 (0.66-1.34)	1.18 (0.87-1.59)	1.19 (0.88-1.61)	0.96 (0.73-1.25)	0.96 (0.73-1.26)
Schizophrenia/psychoses	3.17 (1.67-6.03)	3.18 (1.67-6.06)	3.42 (2.16-5.42)	3.33 (2.1-5.27)	1.19 (0.83-1.7)	1.21 (0.85-1.74)
Other mental disorders	1.22 (1.03-1.45)	1.22 (1.03-1.44)	1.29 (1.12-1.49)	1.29 (1.12-1.49)	1.01 (0.89-1.14)	1.02 (0.9-1.15)
Somatic disorders (ref: no)						
Musculoskeletal disorders	1.68 (1.56-1.81)	1.69 (1.57-1.82)	2.04 (1.9-2.19)	2.03 (1.89-2.19)	1.08 (1.01-1.16)	1.09 (1.01-1.17)
Other somatic disorders	1.54 (1.49-1.6)	1.54 (1.48-1.59)	1.84 (1.77-1.92)	1.83 (1.76-1.9)	1.14 (1.1-1.19)	1.14 (1.1-1.19)
Occupational branch						
Construction	*Not included*	*Reference*	*Not included*	*Reference*	*Not included*	*Reference*
Manufacturing	*Not included*	0.92 (0.85-0.98)	*Not included*	0.78 (0.72-0.85)	*Not included*	1.05 (0.98-1.13)
Trade and communication	*Not included*	0.64 (0.60-0.69)	*Not included*	0.73 (0.68-0.79)	*Not included*	0.65 (0.61-0.7)
Financial and business services	*Not included*	0.77 (0.71-0.83)	*Not included*	0.66 (0.61-0.73)	*Not included*	0.94 (0.86-1.02)
Education and research	*Not included*	0.75 (0.68-0.82)	*Not included*	0.87 (0.79-0.97)	*Not included*	0.68 (0.62-0.76)
Health and social care	*Not included*	0.7 (0.66-0.76)	*Not included*	0.98 (0.91-1.06)	*Not included*	0.53 (0.49-0.58)
Other	*Not included*	0.77 (0.72-0.83)	*Not included*	0.78 (0.72-0.84)	*Not included*	0.83 (0.77-0.89)

CI: confidence interval. Zero inflated binomial regression analysis was used to estimate the odds of not being risk at the outcome and the count of the outcomes during the follow-up. The reciprocal values of the odds ratios and 95% confidence interval is presented in the table.

Supplement table S6. Associations between Attention Deficit Hyperactivity Disorder (ADHD) diagnosis with and without comorbid mental disorder and labour market marginalization (LMM). Incidence rate ratios (IRR) of LMM days and Odds Ratios (OR) of probability of >0 LMM days

	**ADHD without mental comorbidities**		**ADHD with mental comorbidities**	
Predictors	*OR (95% CI)*	*IRR (95% CI)*	*OR (95% CI)*	*IRR (95% CI)*
Exposure				
No ADHD	Reference	Reference	Reference	Reference
ADHD	2.28 (2.04 -2.54)	1.47 (1.38 – 1.56)	4.52 (4.08-5)	2.42 (2.32 – 2.52)
Age groups				
19-24	Reference	Reference	Reference	Reference
25-29	1.07 (1.0 - 1.15)	1.35 (1.29 – 1.41)	1.13 (1.07-1.18)	1.34 (1.29 – 1.38)
Gender				
Female	Reference	Reference	Reference	Reference
Male	0.72 (0.68-0.78)	1.14 (1.09 – 1.20)	0.7 (0.67 - 0.74)	1.11 (1.08 – 1.15)
Years of education				
0-9 years	Reference	Reference	Reference	Reference
10-12 years	0.62 (0.56 - 0.68)	0.74 (0.70 – 0.78)	0.67 (0.62 - 0.72)	0.80 (0.77 – 0.83)
> 12 years	0.45 (0.40 - 0.50)	0.62 (0.57 – 0.67)	0.47 (0.43 - 0.51)	0.67 (0.63 – 0.70)
Country of origin				
Sweden	Reference	Reference	Reference	Reference
Nordic countries	0.85 (0.56-1.29)	0.90 (0.68 – 1.19)	0.96 (0.73-1.26)	1.07 (0.90 – 1.28)
EU	1.25 (1.02-1.55)	1.27 (1.10 – 1.46)	1.17 (1.01-1.37)	1.36 (1.23 – 1.51)
Outside EU	1.56 (1.41-1.73)	1.32 (1.23 – 1.41)	1.75 (1.62-1.88)	1.31 (1.25 – 1.38)
Family status				
Single without children	Reference	Reference	Reference	Reference
Single with children	1.55 (1.20-2.00)	1.21 (1.06 – 1.38)	1.39 (1.18-1.65)	1.08 (0.99 – 1.17)
Married or cohabitant without children	1.19 (0.99-1.42)	1.16 (1.03 – 1.31)	1.29 (1.14-1.45)	1.02 (0.94 – 1.10)
Married or cohabitant with children	0.97 (0.88-1.07)	0.93 (0.88 – 0.99)	1.03 (0.97-1.11)	0.90 (0.87 – 0.94)
Region				

Large cities	Reference	Reference	Reference	Reference
Medium-sized cities	1.28 (1.2-1.38)	1.04 (0.99 – 1.09)	1.29 (1.23-1.35)	1.03 (0.99 – 1.06)
Rural areas	1.55 (1.43-1.67)	1.04 (0.98 – 1.09)	1.45 (1.37-1.53)	1.01 (0.98 – 1.05)
Income quantile				
First quantile	Reference	Reference	Reference	Reference
second quantile	0.93 (0.86-1.01)	1.07 (1.01 – 1.13)	0.97 (0.91 - 1.03)	0.95 (0.91 – 0.98)
third quantile	0.77 (0.70 - 0.84)	0.87 (0.82 – 0.92)	0.79 (0.75 - 0.85)	0.83 (0.79 – 0.86)
fourth quantile	0.50 (0.45 - 0.55)	0.67 (0.63 – 0.72)	0.51 (0.48 - 0.55)	0.67 (0.64 – 0.70)
Blue/White collar worker				
Blue collar	Reference	Reference	Reference	Reference
White collar	0.81 (0.74 - 0.88)	0.98 (0.92 – 1.05)	0.79 (0.75 - 0.85)	0.93 (0.89 – 0.98)
Other	1.08 (0.98 - 1.18)	0.97 (0.92 – 1.03)	1.05 (0.98-1.12)	1.04 (1.00 – 1.09)
Type of work				
Employed	Reference	Reference	Reference	Reference
Self-employede	0.43 (0.35 - 0.51)	0.88 (0.76 – 1.02)	0.44 (0.39 - 0.50)	0.98 (0.89 – 1.08)
SA before baseline (ref no)				
Yes	8.98 (7.56 -10.68)	1.56 (1.47 – 1.66)	9.35 (8.29-10.54)	1.65 (1.59 – 1.71)
Somatic disorders (Ref: no)				
Musculoskeletal disorders	1.72 (1.50-1.96)	1.08 (1.01 – 1.16)	1.69 (1.54-1.85)	1.14 (1.09 – 1.20)
Other somatic disorders	1.54 (1.45-1.63)	1.05 (1.01 – 1.10)	1.57 (1.50 - 1.64)	1.04 (1.01 – 1.07)
Branch				
Construction	Reference	Reference	Reference	Reference
Manufacturing	0.89 (0.79 - 1.01)	1.05 (0.97 – 1.14)	0.93 (0.85-1.02)	1.05 (0.99 – 1.11)
Trade and communication	0.67 (0.60 - 0.74)	0.95 (0.88 – 1.02)	0.64 (0.59 -0.69)	0.96 (0.91 – 1.02)
Financial and business services	0.77 (0.67 - 0.87)	1.14 (1.05 – 1.25)	0.77 (0.70 - 0.85)	1.04 (0.98 – 1.11)
Education and research	0.76 (0.65 - 0.89)	0.92 (0.83 – 1.03)	0.74 (0.66 - 0.83)	0.94 (0.87 – 1.02)
Health and social care	0.69 (0.61 - 0.77)	0.93 (0.86 – 1.01)	0.72 (0.66 - 0.79)	0.96 (0.91 – 1.02)
Other	0.76 (0.68 - 0.86)	1.00 (0.93 – 1.08)	0.78 (0.72 - 0.85)	1.04 (0.99 – 1.10)

CI: confidence interval. Zero inflated binomial regression analysis was used to estimate the odds of not being risk at the outcome and the count of the outcomes during the follow-up. The reciprocal values of the odds ratios and 95% confidence interval is presented in the table.
